# Rhabdomyolysis associated with *Ophioglossum vulgatum* consumption: a case series and safety implications

**DOI:** 10.3389/fphar.2026.1807082

**Published:** 2026-04-22

**Authors:** Xiaorong Li, Lindan Liao

**Affiliations:** 1 Department of Urology, The First People’s Hospital of Neijiang, Neijiang, Sichuan, China; 2 Department of Clinical Pharmacy, The First People’s Hospital of Neijiang, Neijiang, Sichuan, China

**Keywords:** adverse reaction, herbal medicine, Ophioglossum vulgatum, rhabdomyolysis, yi zhi jian

## Abstract

**Background:**

*O. vulgatum* (*Ophioglossum vulgatum*, commonly known as “Yi Zhi Jian”) is a traditional herbal medicine and food ingredient widely used for its heat-clearing and detoxifying properties and generally considered safe. However, its potential for severe muscular toxicity, particularly rhabdomyolysis, has not been systematically reported.

**Case Presentation:**

A retrospective case series analysis was conducted on 10 patients admitted to the First People’s Hospital of Neijiang City from 2024 to 2025 with *O. vulgatum* poisoning complicated by rhabdomyolysis. All patients ingested *O. vulgatum* for the purpose of “stewing soup”, with a median latency period of 7 h. The predominant symptoms were severe myalgia (100%), fatigue (50%), and paresthesia (20%). The median peak creatine kinase (CK) level was 4099 U/L (range 2,427–17250 U/L). Acute kidney injury (AKI) complicated the course in one patient (10%). All patients immediately discontinued the herbal intake and received supportive treatment including aggressive hydration and urine alkalinization. The median hospital stay was 6 days. At the last follow-up, all patients survived, with complete resolution of muscular symptoms and recovery of renal function. The causal relationship between rhabdomyolysis and *O. vulgatum* was assessed as “probable” for all ten patients according to the WHO-UMC scale.

**Conclusion:**

This is the first case series indicating an association between *O. vulgatum* consumption and severe rhabdomyolysis. Clinicians and the public should be aware of this risk and should avoid excessive or unconventional use. A detailed history of herbal medicine use is recommended for patients presenting with unexplained myalgia and elevated CK levels.

## 1 Introduction

*Ophioglossum vulgatum* (*Ophioglossum vulgatum*) is an ancient fern belonging to the family Ophioglossaceae, primarily distributed across Europe, Asia, and the Americas. In China, its distribution is relatively concentrated in regions such as Yunnan, Sichuan, Guangxi, and Taiwan. Known by various folk names such as Yizhijian, Shexucao, Maoduncao, it has been used in traditional Chinese medicine theory for its properties of clearing heat, cooling blood, detoxifying, and alleviating pain. It is traditionally used for conditions such as cough due to lung heat, lung abscess, hemoptysis in pulmonary tuberculosis, high fever and convulsions in children, red and swollen eyes, stomach pain, furuncles and abscesses, snake or insect bites, and traumatic swelling and pain (Clericuzio M et al., 2014; Yang D et al., 2019). Modern pharmacological studies have confirmed that extracts from plants of the Ophioglossaceae family exhibit anti-inflammatory, antioxidant, and antimicrobial activities (Zhu X et al., 2025; Wan CX et al., 2011; Dong JW et al., 2016). However, rigorously designed human clinical trials confirming efficacy and safety are lacking despite these preclinical data. In recent years, in line with the concept of “medicinal and edible homology”, *O. vulgatum* has frequently been used in soups for its purported health benefits, reinforcing its public perception as safe and low-toxic. However, the safety profile of many traditional herbs, including *O. vulgatum*, largely rests on historical empirical evidence rather than modern toxicological evaluation (Posadzki P et al., 2013). Common adverse reactions associated with traditional Chinese herbal medicines include hepatorenal and neurotoxicity. Highly representative herbs such as Aristolochia species, Polygonum multiflorum, and Aconitum species possess potent toxicity, warranting extreme caution in clinical application (Yang B et al., 2018). In contrast, herbs perceived as low-toxicity may receive insufficient safety scrutiny, creating a potential blind spot. This knowledge gap could raise public health concerns, particularly given its widespread oral consumption. To date, literature linking *O. vulgatum* to specific adverse reactions remains scarce, consistent with its traditional characterization as low-toxicity. However, this stereotype may obscure its potential for severe toxicity—such as rhabdomyolysis, a condition rarely associated with folk medicine use.

Rhabdomyolysis is a clinical syndrome characterized by acute destruction of striated muscle cells, leading to the release of intracellular contents into the systemic circulation. This can result in life-threatening complications, including severe electrolyte imbalances, AKI, and multiple organ dysfunction (Kuok and Chan, 2025). Drug-induced rhabdomyolysis has garnered increasing attention with statins being the most recognized class (Norman DJ et al., 1988). However, an expanding variety of herbal preparations can induce similar pathological reactions through diverse and often inadequately understood mechanisms. They may involve direct mitochondrial toxicity (e.g., Tripterygium wilfordii), electrolyte disturbances (e.g., Glycyrrhiza-induced hypokalemia), or misuse (e.g., Lardizabalaceae replaced by Caulis aristolochiae manshuriensis) (Lu S et al., 2026; Attout H et al., 2020; Yang B et al., 2018). Several dietary supplements, such as Tribulus terrestris, red yeast rice, black cohosh, and ephedra, have been reported to cause rhabdomyolysis, often through drug-herb interactions (Huff R et al., 2024; Dernbach MR et al., 2024; Prasad GV et al., 2002; Stahl CE et al., 2006).

Systematic reports linking *O. vulgatum* to rhabdomyolysis are exceedingly rare despite its widespread use. This disparity between widespread public use, partial pharmacological evidence of efficacy, and lack of formal safety data underscores an urgent need for clinical investigation. The absence of documented myotoxicity in major pharmacovigilance databases does not equate to safety, especially given the variability in herb preparation, dosage, and individual susceptibility. We conducted a retrospective analysis of 10 cases of rhabdomyolysis following *O. vulgatum* ingestion treated at our institution. This study aims to systematically delineate the clinical presentation, identify potential risk factors, describe therapeutic interventions, and evaluate outcomes associated with this under-recognized toxicity.

## 2 Case series

### 2.1 General information

This case series retrospectively analyzed ten patients with poisoning following the ingestion of *O. vulgatum*. The inclusion criteria were as follows: (1) a clear history of isolated *O. vulgatum* ingestion without co-administration of other herbal substances; (2) symptom onset within 24 h of ingestion; (3) presence of muscular symptoms such as myalgia, weakness; (4) peak serum creatine kinase (CK) > 1000 U/L (>5 times the upper limit of normal); (5) exclusion of other common causes of rhabdomyolysis (e.g., strenuous exercise, trauma, statin use, severe infection, viral illness, acute thrombotic events, autoimmune myopathies, and toxic exposures such as carbon monoxide, alcohol, snake venom, and excessive medication). Baseline characteristics and clinical symptoms are presented in Table 1. *O*. *vulgatum* exposure characteristics of individual patients was presented in Table 2. Major adverse events were graded according to the Common Terminology Criteria for Adverse Events (CTCAE) version 5.0 and the results are summarized in Table 3. Clinical management and outcomes are provided in Table 4. All patients recovered fully; none required intensive care unit admission. However, one patient developed acute kidney injury (AKI) as a complication of rhabdomyolysis. Using the to the World Health Organization-Uppsala Monitoring Centre (WHO-UMC) scale, the causal relationship between *O. vulgatum* ingestion and rhabdomyolysis was assessed as “probable” for all ten cases based on clear temporal association and reasonable exclusion of alternative causes (Manjhi PK et al., 2024).

**TABLE 1:** Demographic characteristics of patients and clinical symptoms.

Case	Sex	Age (year)	Comorbidities	Latency period	Myalgia				
					Whole body	Limbs	Waist	Abdomen	Chest/Shoulder/Back
1	F	49	5	5 H	1		1	1	
2	M	74	6	1 D	1				
3	F	49	-	9 H				1	
4	F	45	8	9 H					1
5	F	80	1,2,3	3 H		1			
6	F	67	1,2,5	5 H		1			
7	F	55	4	1 D	1				
8	F	55	-	5 H	1		1		1
9	M	49	1	1 H					1
10	F	61	1,7	1 D	1				1

Fatigue	Exertional dyspnea	Dizziness	Nausea and vomiting	Decreased appetite	Chest tightness	Paresthesia	Myoclonus	Melena	Tremor
1		1	1						
1	1			1					1
						1	1		
									
						1			
1									
1									
1					1			1	
									
									

M, male; F, female; H, hour(s); D, day; Comorbidities, 1, diabetes; 2, hypertension; 3, cerebral infarction; 4, hyperthyroidism; 5, hypothyroidism; 6, chronic obstructive pulmonary disease; 7, bronchial asthma; 8, cancer.

**TABLE 2:** *Ophioglossum vulgatum* exposure characteristics of individual patients.

Case	1	2	3	4	5	6	7	8	9	10
Source	Market	Market	Market	Market	Market	Market	Market	Market	Market	Market
Part used	Whole plant	Whole plant	Whole plant	Whole plant	Whole plant	Whole plant	Whole plant	Whole plant	Whole plant	Whole plant
Form	Dried	Fresh	Fresh	Fresh	Fresh	Fresh	Fresh	Fresh	Fresh	Fresh
Dosage	100 g	50 g	100 g	100 g	50 g	50 g	100–150 g	NQ	NQ	50 g
Decoction time	1 h	1 h	0.5–1 h	1 h	2 h	2 h	1.5 h	1.5 h	1.5 h	0.5 h
Preparation method	Stew	Stew	Stew	Stew	Stew	Stew	Stew	Stew	Stew	Stew

NQ, not quantified, g, grams, h, hour (s).

**TABLE 3:** Classification of major adverse reactions.

Preferred term	All grades^a^		Grade ≥3^a^	
	n	%	n	%
Myalgia	10	100	10	100
Creatine kinase increased	10	100	10	100
Rhabdomyolysis	10	100	10	100
Fatigue	5	50	5	50
Acute kidney injury	1	10	1	10
Alanine aminotransferase increased	7	70	2	20
Aspartate aminotransferase increased	10	100	6	60
Alkaline phosphatase increased	1	10	0	0

^a^ Toxicities were graded according to CTCAE v5.0 criteria.

**TABLE 4:** Clinical management and outcomes of patients.

Case	LHS (days)	CK peaking (U/L)	Mb peaking (ng/mL)	ALT peaking (U/L)	AST peaking (U/L)	AKI	Time to CK normalization (Days)^#^	Therapeutic interventions	Renal function recovery	Outcomes
1	6	2,991	>3,000	58	95	N	4	Normal saline; vitamin C; tiopronin	Normal	Recovery
2	25	6,414	59^*^	251	855	Y	5	Normal saline, sodium bicarbonate; furosemide; glutathione, magnesium isoglycyrrhizinate; meglumine adenosine cyclophosphate	Full-recovery	Recovery
3	5	3,225	>3,000	45	93	N	6	Normal saline; furosemide; tiopronin; meglumine adenosine cyclophosphate, coenzyme Q10	Normal	Recovery
4	2	17,250	>3,000	132	343	N	7	Magnesium sulfate (oral powder); sodium bicarbonate, compound sodium chloride injection; glutathione, tiopronin	Normal	Recovery
5	5	2,935	>3,000	36	103	N	572 U/L at discharge	Normal saline, sodium potassium magnesium calcium injection; tiopronin	Normal	Recovery
6	15	4,973	>3,000	34	250	N	5	Dexamethasone; normal saline; mecobalamin, vitamin B_1_	Normal	Recovery
7	6	2,427	>3,000	76	198	N	3	Sodium bicarbonate, normal saline; methylprednisolone; magnesium isoglycyrrhizinate, polyene phosphatidylcholine	Normal	Recovery
8	11	3,203	2,402	49	108	N	6	Normal saline; glutathione; vitamin C	Normal	Recovery
9	5	6,352	2,148	64	137	N	422 U/L at discharge	Medicinal charcoal, magnesium sulfate (oral powder); sodium bicarbonate, compound sodium chloride injection; furosemide; polyene phosphatidylcholine	Normal	Recovery
10	7	7,650	>3,000	28	136	N	515 U/L at discharge	Normal saline; meglumine adenosine cyclophosphate; vitamin C	Normal	Recovery

LHS, length of hospital stay; CK, creatine kinase; Mb, Myoglobin; ALT, alanine aminotransferase; AST, aspartate aminotransferase; AKI, acute kidney injury; Y, yes; N, no. Note: *Case 2 exhibited a dissociation phenomenon with normal myoglobin but significantly elevated CK, and serum creatinine. This is mainly attributed to the short half-life of myoglobin and the delayed presentation, while advanced age and individual susceptibility may have exacerbated the mismatch between renal impairment and myoglobin levels. #Normal range for CK, was defined as 8–190 U/L and discharge values were considered normal if they fell within this range.

### 2.2 Detailed description of representative cases

Three representative cases were selected, and their details are reported as follows.

#### 2.2.1 Case 2

A 74-year-old male with a history of normal liver and kidney function presented with generalized myalgia, hand tremors, exertional dyspnea and fatigue. Symptoms began 1 day after he consumed approximately 50 g of fresh whole *O. vulgatum* plants purchased from a local market, which had been stewed with duck for about 1 hours at home. Upon admission, multiple abnormal laboratory findings were noted: alanine aminotransferase (ALT) 251 U/L (5–40), aspartate aminotransferase (AST) 855 U/L (5–40), alkaline phosphatase (ALP) 160 U/L (45–125), lactate dehydrogenase (LDH) 1257 U/L (100–350), creatinine 233.5 μmol/L (70–115), and creatine phosphokinase (CK) 6414 U/L (8–190). Notably, serum myoglobin was only 59 ng/mL (20–72) despite the presence of acute kidney injury and marked CK elevation. Treatment included aggressive intravenous hydration (normal saline), urine alkalinization (sodium bicarbonate), liver support (glutathione and magnesium isoglycyrrhizinate), myocardial nutritional support (meglumine adenosine cyclophosphate), and diuretic therapy (furosemide) to maintain adequate urine output. The patient’s symptoms, including myalgia, gradually resolved. CK, creatinine, ALT, and AST returned to normal levels on days 5, 15, 12, and 15 after their respective peaks, with complete recovery of renal function. The patient was discharged after 25 days of hospitalization. At the 12-month follow-up, liver and kidney function indices were within normal ranges, and no residual symptoms were reported.

#### 2.2.2 Case 4

A 45-year-old female was admitted due to low back pain and referred pain in the left shoulder lasting approximately 7 h. The onset of symptoms occurred about 9 h after consuming about 100 g of fresh whole *O. vulgatum* plants purchased from a local market which had been stewed with duck for about 1 hours. Laboratory tests upon admission revealed: ALT 132 U/L (5–40), AST 343 U/L (5–40), LDH 800 U/L (100–350), CK markedly elevated at 17,250 U/L (8–190), myoglobin (Mb) > 3,000 ng/mL (20–58), normal high-sensitivity troponin and creatinine, and urine occult blood (3+). The patient was diagnosed with food poisoning, rhabdomyolysis, and abnormal liver function. Supportive treatment included intravenous hydration (compound sodium chloride injection), urine alkalinization (sodium bicarbonate), catharsis (magnesium sulfate oral powder) to promote toxin elimination, and liver support (glutathione and tiopronin). The patient reported improvement in symptoms, particularly low back pain, after 2 days and requested discharge. At a follow-up visit 5 days post-discharge, creatinine and cardiac enzyme profiles had returned to normal, while ALT and AST had significantly decreased to 80 U/L and 41 U/L, respectively. Approximately 2 months after discharge, follow-up tests showed normal liver and kidney function and cardiac enzymes, with no residual symptoms reported.

#### 2.2.3 Case 6

A 67-year-old female was admitted to the hospital due to muscle pain and weakness in all four limbs for 6 h. The symptoms occurred 5 h after she consumed approximately 50 g of fresh whole *O. vulgatum* plants purchased from a local market which had been stewed with duck for about 2 hours. Laboratory tests upon admission revealed: CK 4973 U/L (8–190), Mb > 3,000 ng/mL (25–58), urine occult blood (3+), ALT 34 U/L (5–40), AST 250 U/L (5–40), and LDH 822 U/L (100–350). Electromyography (EMG) indicated mild polyneuropathy in the lower limbs and mild neuropathy in the upper limbs. Supportive treatment included intravenous hydration with normal saline and neurotrophic therapy (mecobalamin and vitamin B_1_). Due to clinical suspicion of an inflammatory component, dexamethasone was administered for its anti-inflammatory effects, although this indication remains unconfirmed (see Discussion). The patient’s limb pain and weakness gradually improved. Both CK and AST returned to normal levels on the fifth day after their respective peaks. The patient was discharged after 15 days of hospitalization with no complaints. Physical examination revealed normal muscle strength and no neurological abnormalities. At the 12-month follow-up, liver and kidney function tests were normal, and no residual symptoms were reported.

## 3 Discussion

This study represents the first systematic report of 10 cases of rhabdomyolysis associated with *O. vulgatum* consumption. These findings challenge the traditional perception of this “medicinal and edible” plant as inherently safe, revealing its potential for severe myotoxicity and underscoring the need for heightened safety scrutiny of such botanicals.

### 3.1 Clinical features and organ involvement patterns

This case series demonstrated widespread, severe, and potentially multi-organ involvement (Tables 1, 3). All patients experienced grade ≥3 (severe) myalgia, CK elevation, and rhabdomyolysis per CTCAE criteria. Notably, toxic manifestations extended beyond the muscular system: AST elevation occurred in all patients (60% grade ≥3), and ALT elevation occurred in 70%. This raises a critical question: does the hepatic involvement reflect secondary release from muscle injury, or direct hepatotoxicity?

Analysis of liver enzyme profiles provides valuable insights. In three patients (Cases 5, 6, and 10), ALT remained normal while AST was elevated, yielding high AST/ALT ratios (2.9–7.4)—a pattern typical of a predominant muscular source of AST, indicating minimal hepatic involvement. However, in the remaining seven patients, ALT was significantly elevated. Critically, ALT levels in Cases 2 and 4 were 251 U/L and 132 U/L, respectively—levels that cannot be explained by muscle breakdown alone, suggesting the presence of hepatocellular injury. Cases 1, 3, 7, 8, and 9 also showed mild ALT elevations (45–76 U/L), supporting direct liver involvement. This coexistence of purely myopathic and mixed hepato-myopathic patterns suggests that *O. vulgatum*’s components may possess broad cytotoxic effects capable of directly damaging both skeletal muscle and hepatocytes, with individual susceptibility potentially determining the clinical phenotype.

Grade ≥3 AKI occurred in one patient (10%), typically a consequence of myoglobinuric tubular obstruction following rhabdomyolysis, though direct nephrotoxicity cannot be excluded. The short median latency period (7 h) points to rapid and potent toxin action.

### 3.2 A clinically important biochemical dissociation

Case 2 presented a significant dissociation: despite normal serum myoglobin (59 ng/mL), the patient exhibited marked CK elevation (6414 U/L) and AKI. This may be primarily attributable to the extremely short serum half-life of myoglobin (2–3 h) and the delayed presentation (24 h post-ingestion). Furthermore, age-related decline in renal reserve and individual susceptibility may have exacerbated the mismatch. This case serves as an important clinical reminder: a single normal myoglobin measurement does not rule out rhabdomyolysis or its renal complications. Assessment should integrate dynamic CK changes, time from symptom onset, and patient baseline characteristics.

### 3.3 Potential risk factors and mechanistic clues

All cases occurred in the context of routine culinary use (stews with duck), suggesting that toxic reactions can occur even with conventional preparation and dosage, implicating unrecognized individual susceptibility. All patients had ingested *O. vulgatum* alone without concurrent use of other herbal products or high-risk medications such as statins, although unrecognized exposures cannot be completely ruled out. The decoction time was clustered around 0.5–2 h. The cohort had a median age of 55 years, and most patients had comorbidities such as hypertension and diabetes, which may impair toxin metabolism and clearance and increase susceptibility to multi-system toxicity (Nguyen KA et al., 2018).

The consistently short latency (1 h–1 day) distinguishes this toxicity from the delayed-onset pattern typical of statin-induced myopathy (Rätz Bravo AE et al., 2005), suggesting a more direct insult to the muscle cell than a slow metabolic dysregulation. Compared to other herb-induced rhabdomyolysis—such as that caused by Tripterygium wilfordii, Glycyrrhiza species or dietary supplements—the rapid onset in our series appears distinctive. This contrasts with the sub-acute presentations often linked to cumulative toxicities or herb-drug interactions.

While definitive mechanism awaits further study, it is important to emphasize that any mechanistic discussion remains speculative, as no toxicological analysis was available from this case series to support direct evidence. Preliminary clues from the known phytochemistry of *O. vulgatum* have identified components such as flavonoids (isorhamnetin, luteolin), alkaloids (betaine), and minor constituents like saponins and anthraquinones (Feng S et al., 2025). Some of these compound classes have been associated with acute cytotoxicity in other contexts (Reyes-Farias and Carrasco-Pozo, 2019; Ogunyemi B T, 2025). However, these hypotheses cannot be confirmed by our data and are presented only as potential directions for future investigation. The primary contribution of this case series remains the clinical description of an under-recognized association, rather than elucidation of pathogenic mechanisms.

### 3.4 Clinical and public health implications

Though lacking direct toxicological evidence in our study, this case series has some clinical and pharmacovigilance implications. The absence of prior case reports may reflect a lack of awareness among clinicians regarding this condition. The distribution and consumption of *O*. *vulgatum* are primarily concentrated in several provinces of southwestern China. This region-specific consumption pattern also limits the scope of case exposure. This study highlights a potentially significant but overlooked public health issue.

From a clinical practice perspective, physicians should maintain suspicion for herb-induced myopathy when evaluating patients with unexplained myalgia, weakness, or elevated CK. A detailed history of herbal medicine use including medicinal cuisine is essential. Management hinges on early recognition, immediate discontinuation of the causative herb, and aggressive supportive care: intravenous fluid resuscitation and urine alkalinization to prevent myoglobin cast nephropathy. Given the frequent co-occurrence of liver injury, systematic monitoring of hepatic function is warranted. Regarding specific therapies: dexamethasone was administered to two patients with suspected neuropathy based on empirical considerations (anti-inflammatory, targeting neuropathy) rather than evidence-based guidelines. It is important to emphasize that corticosteroid use in rhabdomyolysis lacks evidence-based support. The favorable outcomes in these cases may reflect adequate hydration rather than any benefit from steroids. Given the potential for adverse effects, such use should be approached with extreme caution.

At the public health level, this study underscores a persistent challenge: the widespread misperception that “natural” remedies are inherently safe. This perception often leads to unsupervised use, bypassing potential safety checks. Targeted educational campaigns should highlight that “medicinal and edible” plants contain bioactive compounds capable of serious adverse effects, especially in susceptible individuals. Consumers should be encouraged to discuss herbal use with healthcare professionals, just as they would with conventional medications.

Furthermore, there is a pressing need to strengthen pharmacovigilance for herbal remedies. Healthcare professionals play a pivotal role: they must maintain clinical suspicion and report suspected adverse reactions to national pharmacovigilance centers. Establishing clear reporting pathways and fostering a non-punitive reporting culture are essential for generating the real-world evidence needed to inform regulatory decisions.

### 3.5 Limitations

This study has several limitations. First, as a single-center retrospective analysis, the sample size is limited and selection bias cannot be excluded. Second, botanical verification was not performed. Although the plant’s distinctive arrow-like morphology reduces the likelihood of misidentification, the reliance on patient reports of consuming *O. vulgatum* from local markets precludes definitive species identification, thereby restricting causal conclusions. Furthermore, no toxicological analysis or screening for contaminants (e.g., pesticides, heavy metals, adulterants) was performed. Consequently, the observed effects could be attributed to the plant’s inherent constituents, contaminants, or batch-to-batch variability in active compound concentrations. Although exposure details (parts consumed, quantity, decoction duration, and preparation method) were collected based on patient recall, the precise concentration of active compounds ingested could not be quantified, which limits our ability to establish a dose-toxicity relationship. In summary, due to the absence of botanical identification and toxicological analysis, this study can only describe a temporal association between *O. vulgatum* ingestion and rhabdomyolysis, not establish definitive causation. Future prospective studies incorporating standardized sample collection, phytochemical analysis, and contaminant screening are needed to validate the safety profile and elucidate the mechanisms of toxicity.

## 4 Patient perspective

All patients reported that the muscle pain was quite severe. Most patients indicated that the use of *O. vulgatum* (a decoction of duck cooked with the herb) was common and they had never heard of it causing rhabdomyolysis, let alone expected it to be so severe. Patient 10 stated that she had eaten much less *O. vulgatum* than her family members, but she was the only one who fell ill. She will be more cautious in using herbal remedies in the future.

## Data Availability

The original contributions presented in the study are included in the article/supplementary material, further inquiries can be directed to the corresponding author.
